# A trend analysis of the prevalence of opioid misuse, social support, and suicide attempt among American Indian/Alaska native high school students in New Mexico: 2009–2019 Youth Risk Resiliency Survey (YRRS)

**DOI:** 10.1186/s12889-022-12764-2

**Published:** 2022-02-21

**Authors:** Daniel Opoku Agyemang, Erin Fanning Madden, Kevin English, Kamilla L. Venner, Handy Rod, Tejinder Pal Singh, Fares Qeadan

**Affiliations:** 1 University of Utah School of Medicine, Department of Family and Preventive Medicine, Salt Lake City, UT USA; 2 Wayne State University, Department of Family Medicine and Public Health Sciences, Detroit, MI USA; 3 Albuquerque Area Southwest Tribal Epidemiology Center, Albuquerque, NM USA; 4 University of New Mexico, Department of Psychology,, Center On Alcohol, Substance use, And Addiction (CASAA), Albuquerque, NM USA; 5 Parkinson School of Health Sciences and Public Health, Loyola University Chicago, Maywood, IL USA

**Keywords:** Suicide attempt, Opioid misuse, American Indian, Alaska Native, Reservation

## Abstract

**Background:**

American Indian and Alaska Native (AI/AN) youth face stark inequities in opioid misuse, social support, and suicide attempt. This study examined trends in these behavioral measures among AI/AN students in New Mexico (NM).

**Methods:**

Using the NM oversampled Youth Resiliency and Risk Survey (NM-YRRS, 2009 – 2019: odd years), prevalence estimates of opioid misuse, social support (SS), and suicide attempt for AI/AN high school students were generated. Trends over time were assessed via linear regression of weighted proportions according to Peter Armitage. Stratified trends by demographics were also employed.

**Results:**

While the prevalence of suicide attempt did not change significantly over time, it was consistently higher among females (2011–2019), those who misused opioids, received low social support, had a mother with less than high school education, had a C, D, or F for academic performance, and non-straight students relative to their counterparts. In particular, the prevalence of suicide attempt among AI/AN students who reported opioid misuse in 2009 was significantly higher by 25.4% than their counterparts who did not report opioid misuse (35.8% vs. 10.4%.) A significant decreasing trend over time (2009–2017) was observed for opioid misuse (16.1%↓8.8%, *p*-value = 0.0033), including when stratifying by sex (males: 15.9%↓9%, *p*-value = 0.002; females: 16.2%↓8.6%, *p*-value = 0.012). Youth with high maternal education exhibited significant decline in opioid misuse (13.5%↓6.7%, *p*-value = 0.019; 2011–2017.) Opioid misuse increased significantly from 2017 to 2019 (8.8%↑12.9%, *p*-value < 0.0001.) For instance, in 2019 among AI/AN students who reported low social support, opioid misuse was roughly doubled (18.9% vs. 8.5%, *p* < 0.0001), and suicide attempt was tripled (21.3% vs. 7.0%, *p* < 0.0001) compared to students with high social support.

**Conclusion:**

No significant trend was observed for suicide attempt. We observed a significant decreasing trend in opioid misuse between 2009 through 2017 but a significant increase from 2017 to 2019. A higher level of maternal education (college or above), and an A or B school grade performance were protective against both opioid misuse and suicide attempt.

## Introduction

Suicide among youth continues to be a significant challenge to public health in the United States (U.S.) and the world at large [1]. Suicide death rates among persons aged 10–24 have increased by 56% from 2007 through 2017 [2] and by 61.7% (from 6.0 to 9.7 per 100,000) among youth aged 14–18 years between 2009 and 2018 in the U.S [1]. The American Indian and Alaska Native (AI/AN) population is disproportionately affected by the suicide crisis in the U.S. [3, 4]. Suicide is the second leading cause of death among young AI/AN populations [3, 5]. Inequities are even more pronounced among youth and young adults, where the age-adjusted suicide rate for AI/AN populations aged 15–24 is 39.7 per 100,000 compared to the overall U.S. rate of 9.9 per 100,000 population [6]. Recent research has documented a substantial increase in major depression, suicidal thoughts, and attempts among U.S. adolescents and young adults [7]. Among students in grades 9–12 in the U.S. in 2013, 17% of students reported that they seriously considered attempting suicide in the previous 12 months, and 13.6% made a suicide plan [3].

Suicide attempt prevalence in New Mexico has been consistently higher than the national prevalence since 2001 [8]. In 2017, the prevalence of past-year suicide attempt by youth in grades 9-12 was significantly higher in New Mexico (9.9%) compared to the U.S. (7.4%); despite it having decreased significantly from 14.3% in 2007 to 9.9% in 2017 [8].

New Mexico is one of the states with the highest proportion (14.5%) of AI/AN residents in the country [9, 10], and the state experienced an increase in suicide death rates in all age-groups between 1999 and 2017 [5, 11]. Among the 10 – 24 years age group, there was a 50% increase in the suicide deaths rate from 15.5 per 100,000 in 1999 to 23.3 per 100,000 in 2017 [5, 11]. A prior suicide attempt is the strongest risk factor for death by suicide in the general population [8, 12, 13]. Therefore, research on AI/AN youth in New Mexico who have attempted suicide can provide key insights into additional protective and risk factors for suicide mortality.

People with opioid use disorder (OUD) are thirteen times more likely to die by suicide than people without an OUD [14]. The opioid-related overdose death rate among AI/AN populations has increased exponentially from 2.7 per 100,000 in 2000 to 17.0 per 100,000 in 2019, which exceeds the 2019 national rate of 15.2 per 100,000 [15]. Risk factors for suicide attempt among AI/AN youth, include witnessing a suicide or knowing a suicide victim [4], using illicit substances [16], having a psychiatric disorder including depression [17, 18], having experienced physical or sexual abuse [17], reporting perceived discrimination [18], experiencing barriers to care like cultural or social beliefs or lack of care providers [19], and experiencing cognitive factors such as feelings of hopelessness and disconnection from one’s culture [20, 21]. Previous research shows illicit substance use is associated with increased suicide risk for AI/AN youth [16], and research on other U.S. populations shows people with OUD, in particular, are at much greater risk for suicide [14]. However, little is known about how opioid misuse and suicide attempt impact AI/AN youth in specific geographic areas over time, nor how such trends may vary by demographic variables.

Although social network characteristics can be positive or negative factors for behavioral health issues in other young populations [22], the role of social networks in the AI/AN youth population grappling with multiple behavioral health issues is less clear. The structure of adolescent social networks is an essential factor in the etiology of behavior, but research has shown that AI/AN youth have fewer ties at school than non-Hispanic White youth, have fewer reciprocated friendships, have a smaller circle of in-school friends, and membership in less-cohesive personal networks [23]. Studies suggest low social support remains a significant risk factor for suicide among rural AI/AN youth [24]. According to reporting from the Albuquerque Area Southwest Tribal Epidemiology Center, high social support networks at home, school, and the community are associated with decreased odds of suicide among AI/AN high school students in New Mexico [25].

Research that characterizes the prevalence of opioid misuse, social support, and suicide attempts among AI/AN youth, stratified by high school rurality and tribal land location, as well as student demographic traits, is notably lacking. This study’s main objective is to characterize opioid misuse, social support, and suicide attempt over time among New Mexico AI/AN high school students, and how such trends vary by demographic variables.

## Methods

### Data source and design

Six years of data from high school students who completed the New Mexico Youth Risk and Resiliency Survey (NM-YRRS) were obtained from the New Mexico Department of Health (NM-DOH). The NM-YRRS data were from 2009, 2011, 2013, 2015, 2017, and 2019. The NM-YRRS, a state-specific component of the national Youth Risk Behavior Surveillance System (YRBSS), is administered in odd years to assess the health risk behaviors and resiliency factors contributing to teen health in New Mexico. The NM-YRRS uses a two-stage cluster sampling design to produce a representative sample of New Mexico high school students in grades 9–12. The first sampling stage selects schools randomly in proportion to school enrollment size. In the second sampling stage, intact classes of a required subject or entire classes during a required period (e.g., second period) are selected randomly. In schools with small student populations, all students were included in the sample. To provide a more robust and representative AI/AN sample size, the Albuquerque Area Southwest Tribal Epidemiology Center (AASTEC) assisted in oversampling AI/AN youth. Each student survey was weighted to adjust for the nonresponse rate and the distribution of students by grade, sex, and race/ethnicity. This study was determined to be exempt by the University of Utah Institutional Review Board (IRB #137,165). Descriptive statistics were generated using Stata version 16.1 (Stata Corp, 2019) and R Studio (version 3.6.1; R studio, Boston, Massachusetts).

### Measures

Variables included in the analysis were asked in all survey years for consistency and comparability. There were three primary outcomes of interest in this study. The first primary outcome of interest was opioid misuse, and it combined non-zero answers to the following two questions, “*During your lifetime, how many times have you used heroin (also called smack, junk, or China White?*” and “*During the past 30 days, how many times did you use a painkiller to get high, like Vicodin, OxyContin (also Oxy or OC), or Percocet (also called Percs)?*” into a category of ever opioid misuse. The years 2017 and 2019 had an additional question on painkiller use, “*During your life, how many times have you taken prescription pain medicine without a doctor’s prescription or differently than how a doctor told you to use it?*” The additional question was added to generate the variable for opioid misuse for 2017 and 2019 (For justification of combining these variables, see [26]).

The second primary outcome is a construct for social support, which combined answers from eight survey questions in four domains including family, school, community, and peers provided in Table 1. The responses to each support question were put into two categories, “Not true at all or A little true” and “Pretty much true or Very much true.” “Pretty much true or Very much true” is considered a positive response, indicative of positive social support, and was coded as 1. “Not true at all or A little true” is coded as 0, indicative of negative social support. To maintain the integrity of the composite score of social support, missing values for each support question were dropped. A composite score for social support was generated by totaling student responses, leading to a possible range from zero to eight. The variable creation followed previous literature on social support constructs [27]. The final composite scores were then categorized into three levels of social support. Students who had scores of three or less were classified as having “low social support,” students scoring four to six were categorized as having “moderate social support” and finally, students with scores of seven or more were classified as having “high social support”[24].

**Table 1:** Individual Social Support Survey Questions

1. Parent or adult at home is interested in my school work?
2. Parent or adult at home believes I will be a success?
3. Teacher or adult at school listens to me?
4. Teacher or adult believes I will be a success?
5. Adult in the community cares about me?
6. Adult in the community tells me good job?
7. A friend my own age really cares about me?
8. When I am not at home, a parent or guardian knows where I am and who I am with?

The third primary outcome was suicide attempt in the past 12 months. This used answers to the question, “*During the past 12 months, how many times did you actually attempt suicide*,” with possible responses of zero through six or more times. The responses were categorized into a binary variable; “Yes” for non-zero times, and “No” for no attempts.

The stratifying variables included high school rurality (urban/rural) and tribal land (on/off) status, and respondent age, sex, sexual identity, and maternal education. Rurality was a binary variable indicating if the school was in an urban or rural county. Similarly, tribal land was a binary variable indicating if the school was located on or off a tribal land. Age was categorized into four groups: ≤ 14 years, 15 years, 16 years, and 17 + years for consistency across all survey years. Biological sex was binary (male vs. female). Sexual identity was a four-category variable, including heterosexual/straight, bisexual, gay or lesbian, and questioning. Maternal education was categorized into three levels: < high school, high school, and college or above.

### Data analysis

Prevalence estimates for opioid misuse, social support, and suicide attempt with 95% confidence intervals were generated among AI/AN students. For each survey year, the prevalence estimates were stratified over time by rurality, tribal land status, age, sex, sexual identity, and maternal education. The trend over time was assessed via linear regression of weighted proportions against years, according to the method described by Peter Armitage [28].

## Results

### Sample characteristics

New Mexico has 33 counties of which 12 are identified as completely or mostly rural according to the Census Bureau [29]. There are 23 Indian tribes located in New Mexico that make up about 11.0% of its population [30].

A total of 3,641 AI/AN students in New Mexico were included in the 2019 survey data. Sample sizes from the other years in this study (i.e., 2009, 2011, 2013, 2015, and 2017) were similar. In 2019, 12.9% of AI/AN students reported opioid misuse, and 12.6% reported at least one suicide attempt in the past 12 months. About 45.6% of AI/AN students answering the survey were 15 years old or less. Most AI/AN students (67.3%) attended schools in rural areas, and only 13.1% attended schools on tribal land (Table 2). In 2019, almost half of the AI/AN student population reported high social support (49.3%). Among AI/AN students who reported low social support, opioid misuse was roughly doubled (18.9% vs. 8.5%, *p* < 0.0001), and suicide attempt was tripled (21.3% vs. 7.0%, *p* < 0.0001) compared to students with high social support. While the prevalence of opioid misuse is similar among AI/AN males (12.8%) and females (13.0%), suicide attempt was twice as prevalent among females. This study reveals a decreasing prevalence in suicide attempt as age increases (15.0% among those of the age ≤ 14 years old vs. 8.8% among those of the age ≥ 17). We also saw strikingly varied prevalence estimates for both opioid misuse and suicide attempt by sexual identity.

**Table 2:** Characteristics of AI/AN youth participants (NM-YRRS, 2019)

	**Total** ***n* (%**^**1**^**)**	**Opioid Misuse** ***n* (%**^**2**^**)**	**No Opioid Misuse** ***n* (%**^**2**^**)**	***P*-value**	**Suicide attempt** ***n* (%**^**2**^**)**	**No Suicide attempt** ***n* (%**^**2**^**)**	***P*-value**
Total	3,641(100)	469 (12.9)	3172 (87.1)	NA	467 (12.6)	3174 (87.4)	NA
Social Support (SS)							
Low SS	661 (17.7)	142 (18.9)	519 (81.1)	** < 0.0001**	145 (21.3)	516 (78.7)	** < 0.0001**
Moderate SS	1,180(33.0)	175 (16.3)	1,005 (83.7)		191 (16.3)	989 (83.7)	
High SS	1,800 (49.3)	152 (8.5)	1,648 (91.5)		131 (7.0)	1,669 (93.0)	
Age (years)							
≤ 14	772 (20.5)	78 (11.3)	694 (88.7)	**0.04**	107 (15.0)	665 (85.0)	**0.02**
15	905 (25.1)	139 (16.6)	766 (83.4)		138 (14.4)	767 (85.6)	
16	983 (26.4)	139 (13.0)	844 (87.0)		125 (13.1)	858 (86.9)	
≥ 17	981 (28.0)	113 (10.6)	868 (89.4)		97 (8.8)	884 (91.2)	
Sex							
Female	1,903 (50.1)	254 (13.0)	1,649 (87.0)	0.91	309 (16.5)	1,594 (83.5)	** < 0.0001**
Male	1,738 (49.9)	215 (12.8)	1,523 (87.2)		159 (8.7)	1,580 (91.3)	
Grade							
9^th^	1,071 (28.3)	123 (12.0)	948 (88.0)	**0.002**	157 (14.5)	914 (85.5)	**0.01**
10^th^	924 (26.0)	143 (17.7)	781 (82.3)		129(14.0)	795 (86.0)	
11^th^	926 (25.3)	119 (11.1)	807 (88.9)		106 (12.3)	820 (87.7)	
12^th^	701 (20.4)	77 (9.2)	624 (90.8)		68 (7.4)	633 (92.6)	
Academic Performance							
A’s / B’s (high grades)	2,235 (69.9)	237 (10.9)	1,998 (89.1)	**0.0004**	226 (10.3)	2,009 (89.7)	** < 0.0001**
C, D, or F’s (poor grades)	933 (30.1)	171 (17.7)	762 (82.3)		173 (16.8)	760 (83.2)	
Reservation							
Tribal land	696 (13.1)	90 (12.4)	606 (87.6)	0.59	95 (13.8)	601 (86.2)	0.18
Non-tribal land	2,728 (86.9)	358 (13.5)	2,370 (86.5)		330 (11.6)	2,398 (88.4)	
Residency							
Rural	2,561 (67.3)	346 (14.1)	2,215 (85.9)	0.17	329 (12.5)	2,232 (87.5)	0.17
Urban	863 (32.7)	102 (11.7)	761 (88.3)		96 (10.6)	767 (89.4)	
Maternal Education Level							
< High School	530 (16.6)	85 (14.9)	445 (85.1)	0.60	80 (18.3)	450 (81.7)	**0.01**
High School	1,574 (51.5)	192 (12.7)	1,382 (87.3)		200 (12.3)	1,374 (87.7)	
≥ College	963 (31.9)	127 (12.8)	836 (87.2)		110 (10.0)	853 (90.0)	
Sexual Identity							
Heterosexual	2,852 (79.5)	294 (10.6)	2,558 (89.4)	** < 0.0001**	268 (8.7)	2,584 (91.3)	** < 0.0001**
Gay/Lesbian	109 (3.0)	33 (32.5)	76 (67.5)		27 (24.8)	83 (75.2)	
Bisexual	447 (12.7)	99 (19.6)	348 (80.4)		132 (32.5)	315 (67.5)	
Questioning	169 (4.8)	35 (21.1)	134 (78.9)		38 (19.4)	131 (80.6)	

^1^% = column percentage

^2^% = row percentage

### Trend analysis

#### Opioid misuse, social support and suicide attempt over time

**Fig. 1:**
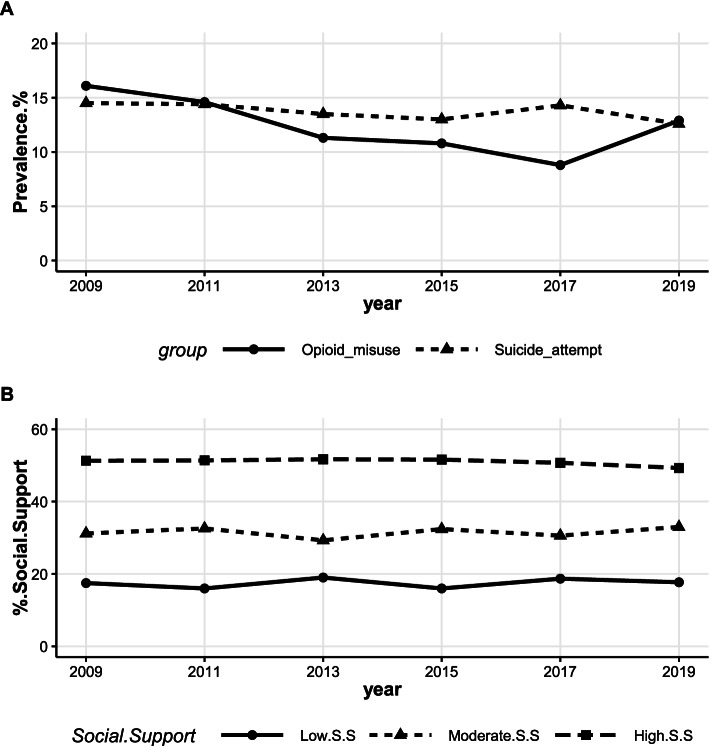
Prevalence of overall suicide attempt, opioid misuse, and social support among AI/AN high school students in New Mexico. Trend: **A** – suicide attempt (t = -1.88, *p*-value = 0.13), opioid misuse (t = -1.85, *p*-value = 0.137 for 2009–2019, and t = -8.60, *p*-value = 0.0033 for 2009–2017), **B** – low social support (t = 0.53 *p*-value = 0.627), moderate social support (t = 0.47, *p*-value = 0.6604), high social support (t = -2.09, *p*-value = 0.1053)

The analysis revealed a steady decline in opioid misuse among AI/AN students in New Mexico from 2009 through 2017 (16.1% ↓ 8.8%; t = -8.60, *p*-value = 0.0033). A sharp increase occurred in the prevalence of opioid misuse from 2017 to 2019 (8.8% ↑ 12.9%–Fig. 1A). The prevalence of opioid misuse was consistently lower than that of suicide attempt from 2011 through 2017 while being comparable in 2019 (12.9% for opioid misuse and 12.6% for suicide attempt).

Overall, high social support remained more common in the sample population than moderate and low social support from 2009 through 2019. Specifically, low, moderate, and high social support prevalence has changed from 17.5%, 31.2%, and 51.3% in 2009 to 17.7%, 33.0%, and 49.3% in 2019 (Fig. 1B). The change in trend over time, from 2009 through 2019, in the prevalence for all levels of social support was not statistically significant (Low Social Support: t = 0.53, *p*-value = 0.627; Moderate Social Support: t = 0.47, *p*-value = 0.6604; and High Social Support: t = -2.09, *p*-value = 0.1053).

Across the study period, the prevalence of suicide attempt among AI/AN high school students declined slightly, yet insignificantly, from 14.5% in 2009 to 13.5% in 2013 to 12.6% in 2019 (t = -1.88, *p*-value = 0.13.) However, the decline was most pronounced from 2017 to 2019 (14.3% ↓ 12.6%–Fig. 1A).

#### Suicide attempt over time by opioid misuse status and social support

**Fig. 2:**
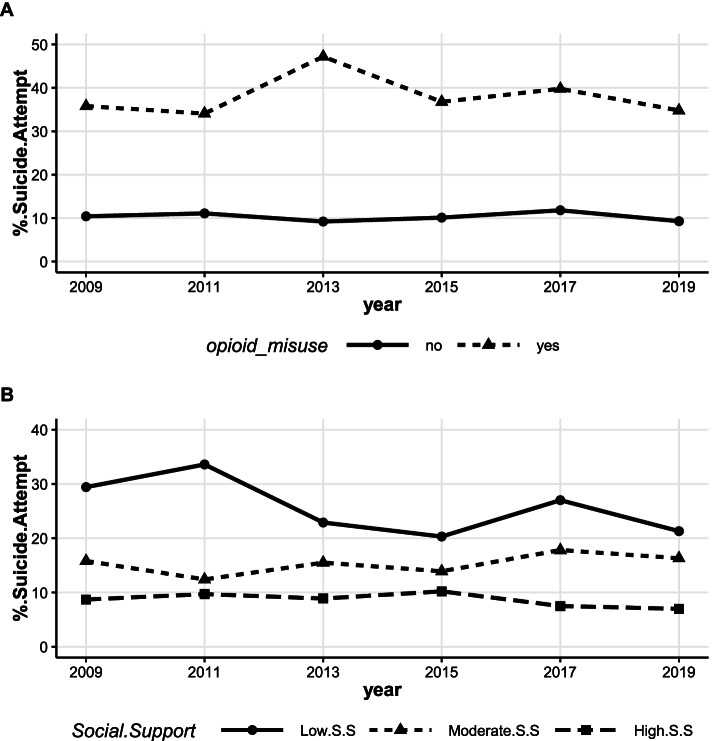
Prevalence of suicide attempt (**A**) by opioid misuse, and social support (**B**) among AI/AN high school students in New Mexico. Trend (**A**): suicide attempt among individuals who report no opioid misuse (t = -0.27, *p*-value = 0.80), suicide attempt among individuals who report opioid misuse (t = 0.04, *p*-value = 0.97). Trend (**B**): low social support (t = -1.71, *p*-value = 0.16), moderate social support (t = 1.10, *p*-value = 0.33), high social support (t = -1.49, *p*-value = 0.21)

Overall, AI/AN students who misused opioids reported higher prevalence of suicide attempt across the years compared to those who did not report opioid misuse. AI/AN students who reported higher social support had relatively lower suicide attempt prevalence than students who reported moderate and low social support (Fig. 2). Specifically, the prevalence of suicide attempt among AI/AN high school students who reported opioid misuse in 2009 was significantly higher by 25.4% than that among AI/AN youth who did not report opioid misuse (35.8% vs. 10.4%). This difference was roughly sustained in 2019 with the prevalence of suicide attempt being 34.8% among those reporting opioid misuse and 9.3% among those who did not misuse opioids (Fig. 2A). There was a spike in the prevalence of suicide attempt among AI/AN who misused opioids in 2013 compared to those who did not misuse opioids (47.2% vs. 9.2%). After the spike in 2013, suicide attempt among AI/AN reporting opioid misuse declined from 47.2% to 34.8% in 2019, whereas suicide attempt rates among AI/AN students who did not misuse opioids remained the same (9.2% to 9.3%). We noted that after 2017, the prevalence of suicide attempt among AI/AN students who reported opioid misuse decreased from 39.8% to 34.8% in 2019. Those who reported no opioid misuse also demonstrated a slight decrease in suicide attempt prevalence from 11.8% in 2017 to 9.3% in 2019, but trend analysis over time revealed no significant change in the prevalence of suicide attempt among those who reported opioid misuse (t = 0.04, *p*-value = 0.97) or those who reported no opioid misuse (t = -0.27, *p*-value = 0.80).

The prevalence of suicide attempt among AI/AN students with high social support was relatively stable over the study period (from 8.7% in 2009 to 7.0% in 2019; t = -1.49, *p*-value = 0.21, Fig. 2B). The data further showed a similar stable trend for those with moderate social support (from 15.8% in 2009 to 16.3% in 2019; t = 1.10, *p*-value = 0.33). Thus, changes in the trend over time for the prevalence of suicide attempt among students with different levels of social support were not statistically significant.

#### Suicide attempt and opioid misuse over time by sex of student and maternal educational status

**Fig. 3:**
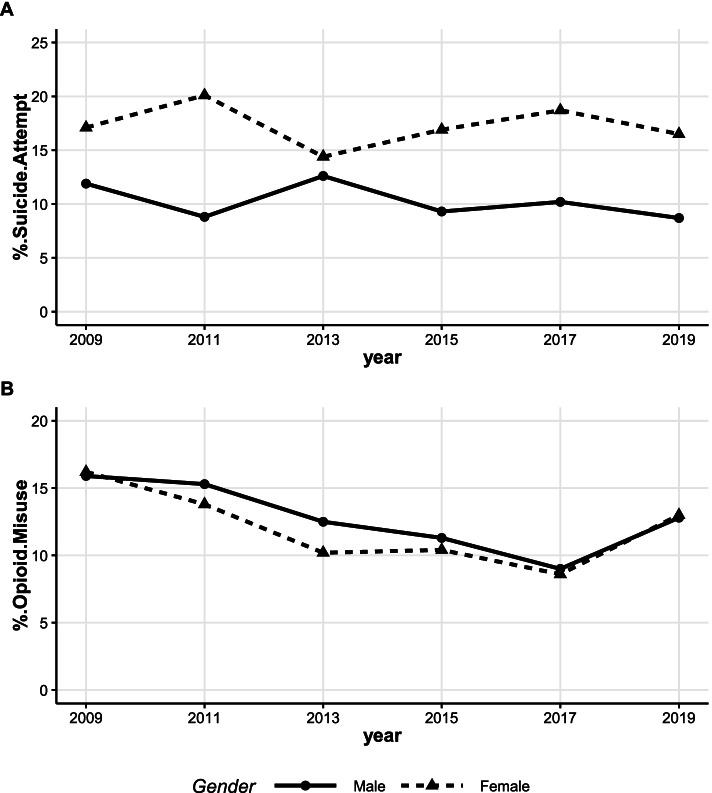
Prevalence of suicide attempt (**A**), opioid misuse (**B**) among AI/AN high school students stratified by sex. Trend: **A**: male (t = -1.12, *p*-value = 0.33), female (t = -0.26, *p*-value = 0.81), **B**: Trend: male (t = -9.97, *p*-value = 0.002 from 2009–2017), female (t = -5.43, and *p*-value = 0.012 from 2009–2017)

Female AI/AN students reported more suicide attempts than their male counterparts; however, male AI/AN students were more likely to report opioid misuse than females in the study period except for 2019, where prevalence of opioid misuse was similar in both male and female AI/AN students. Specifically, from 2009 through 2019, the prevalence of suicide attempt among female AI/AN students was consistently higher than that of males. Though suicide attempt plummeted among female AI/AN students from 20.1% in 2011 to 14.4% in 2013, it rose steadily to 18.7% in 2017 before dropping again to 16.5% in 2019 (Fig. 3A). Suicide attempt among male AI/AN students increased from 8.8% in 2011 to 12.6% in 2013. But there was no statistically significant change in trend of suicide attempt among AI/AN male and female students over time (male: t = -1.12, and *p*-value = 0.33; female: t = -0.26, and *p*-value = 0.81; Fig. 3A). On the other hand, prevalence of opioid misuse demonstrated significant decline for both male and female AI/AN students from 2009 to 2017 (t = -5.43, and *p*-value = 0.012 for females; t = -9.97, and *p*-value = 0.002 for males). However, the prevalence of opioid misuse among male AI/AN students was higher than female AI/AN students over the study period. AI/AN male and female student opioid misuse increased from 9% and 8.6% in 2017 to 12.8% and 13% in 2019, respectively (Fig. 3B).

**Fig. 4:**
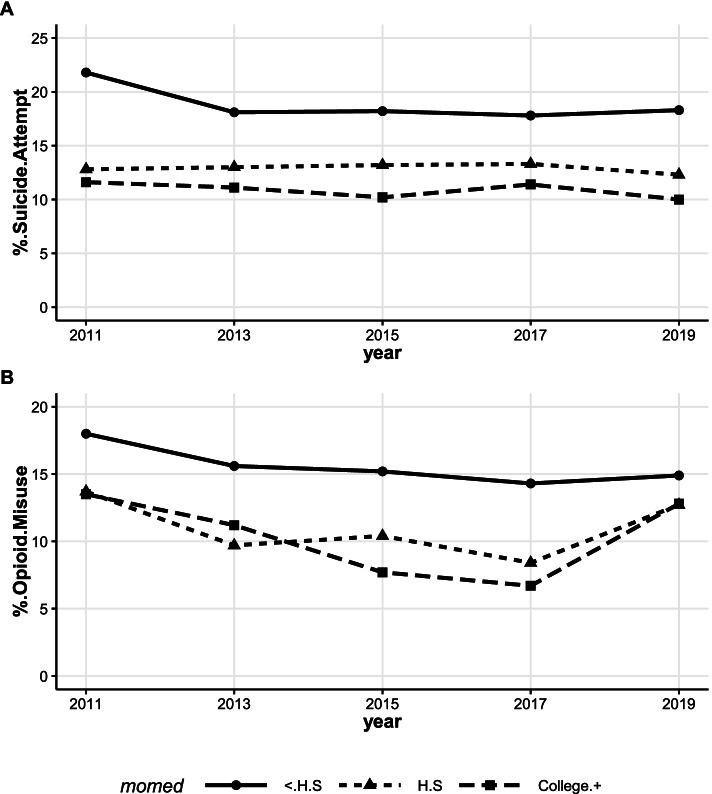
Prevalence of suicide attempt (**A**), opioid misuse (**B**) among AI/AN youth by maternal education. Trend: **A**: < HS (t = -1.67, *p*-value = 0.19), HS (t = -0.50, *p*-value = 0.65), College + (t = -1.43, *p*-value = 0.25). **B**: Trend: < HS (t = -2.61, *p*-value = 0.08), HS (t = -0.43, *p*-value = 0.70), College + (t = -0.56, *p*-value = 0.62, t = -7.18, *p*-value = 0.019 for 2011 to 2017)

AI/AN students in New Mexico with mothers holding less than a high school diploma consistently recorded the highest prevalence of suicide attempts and opioid misuse across all survey years from 2009 through 2019 (Fig. 4A and B). AI/AN students with mothers holding a college degree or higher had the lowest prevalence of both suicide attempt and opioid misuse with the exception of the 2011 to 2013 period for opioid misuse. In these two years, the prevalence of opioid misuse was higher among AI/AN students whose mothers had a college degree, than AI/AN youth who had mothers with high school education. Opioid misuse by AI/AN students with mothers holding a college degree declined significantly from 18.0% in 2011 to 6.7% in 2017 (t = -7.18, *p*-value = 0.019) but increased again in 2019 (Fig. 4B).

#### The prevalence of suicide attempts, opioid misuse, and social support by high school tribal land location and rurality

**Fig. 5:**
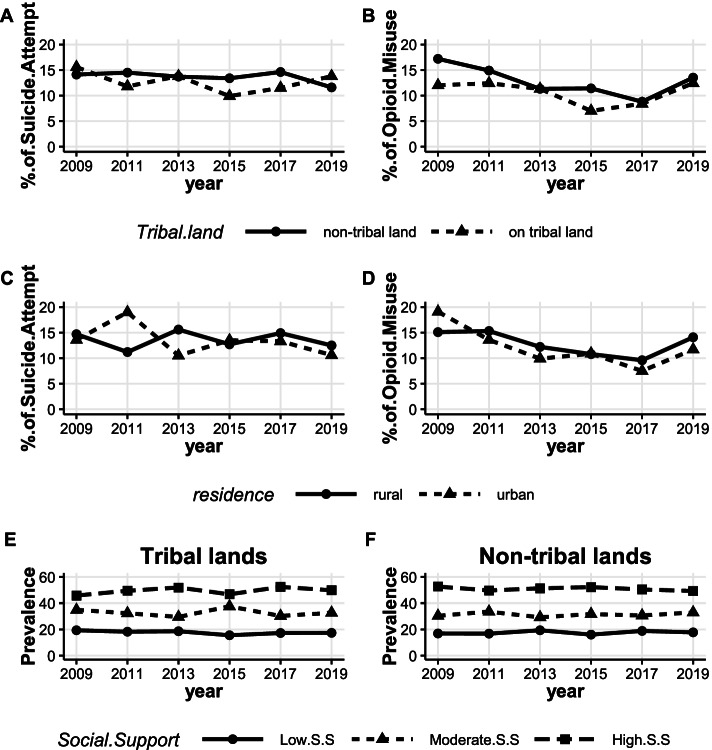
Prevalence of suicide attempt, opioid misuse stratified by high school tribal land status (**A**, **B**) and rurality (**C**, **D**) and the prevalence of social support (**E**, **F**) stratified by tribal land status among AI/AN youth in New Mexico. Trend: **A**: on tribal land (t = -0.75, *p*-value = 0.49), non-tribal land (t = -1.52, *p*-value = 0.20). **B**: on tribal land (t = -0.70, *p*-value = 0.52), non-tribal land (t = -1.75, *p*-value = 0.16 for overall, t = -6.72; *p* = 0.0067 for 2009 to 2017). **C**: rural school suicide attempt (t = -0.18, *p*-value = 0.87), urban school suicide attempt (t = -1.17, *p*-value = 0.31). **D**: rural school opioid misuse (t = -1.26, *p*-value = 0.28 for overall, t = -6.13, *p*-value = 0.0087), urban school opioid misuse (t = -2.14, *p*-value = 0.099 for overall, t = -4.17; *p*-value = 0.0251 for 2009 to 2017). **E**: on tribal lands: Low Social Support (t = -1.63, *p*-value = 0.1786), moderate social support (t = -0.39, *p*-value = 0.7147), high social support (t = 1.11, *p*-value = 0.3292). **F**: non-tribal land: low social support (t = 0.64, *p*-value = 0.5586), moderate social support (t = 0.43, *p*-value = 0.6925), high social support (t = -1.26, *p*-value = 0.2748)

In 2009, suicide attempt prevalence was higher among New Mexico AI/AN students who attended high schools in tribal communities than those who attended schools in non-tribal lands. However, from 2011–2017 the prevalence of suicide attempt and opioid misuse among students who attend schools in non-tribal lands was consistently higher than that of youth who attend schools on tribal lands. In 2019, suicide attempt prevalence was higher in schools that are on tribal lands relative to non-tribal land schools; however, opioid misuse remained higher among AI/AN youth who attended schools in non-tribal lands. No significant change in trend was observed in the prevalence of suicide attempt over time among AI/AN youth who attended high schools either on tribal lands or non-tribal lands (Fig. 5A). The prevalence of opioid misuse declined from 17.2% in 2009 to 8.8% in 2017 (t = -6.72; *p* = 0.0067) among AI/AN youth who attended schools in non-tribal lands (Fig. 5B). However, the prevalence of opioid misuse declined from 12.0% in 2009 to 8.4% in 2017 (t = -2.63; *p* = 0.08) among AI/AN youth who attended schools on tribal lands (Fig. 5B). The data also show that the prevalence of suicide attempt and opioid misuse was consistently higher, from 2013 to 2019, among students attending schools in rural areas (Fig. 5C and D). We observed that the prevalence of opioid misuse among students who attended schools in urban areas declined from 19.1% in 2009 to 7.5% in 2017 (t = -4.17; *p*-value = 0.0251). Similar trend was observed among students who attended schools in rural areas (15.1% ↓ 9.6% from 2009 to 2017; t = -6.13; *p*-value = 0.0087).

The prevalence of low social support was relatively stable among AI/AN students who attend high schools on tribal lands (19.3% in 2009 and 17.4% in 2019). The prevalence of moderate social support increased from 34.9% in 2009 to 37.6% in 2015 among youth who attend high schools on tribal lands. However, the observed increase was not sustained in 2019 (32.7%, Fig. 5E). The prevalence of high social support was higher than moderate and low social support among AI/AN youth who attend schools on both tribal and non-tribal lands (Fig. 5E and F). No significant change in trend was observed over the years for all levels of social support among students who attend high schools either on tribal lands or non-tribal lands.

#### The prevalence of suicide attempt and opioid misuse by sexual identity and academic performance

**Fig. 6:**
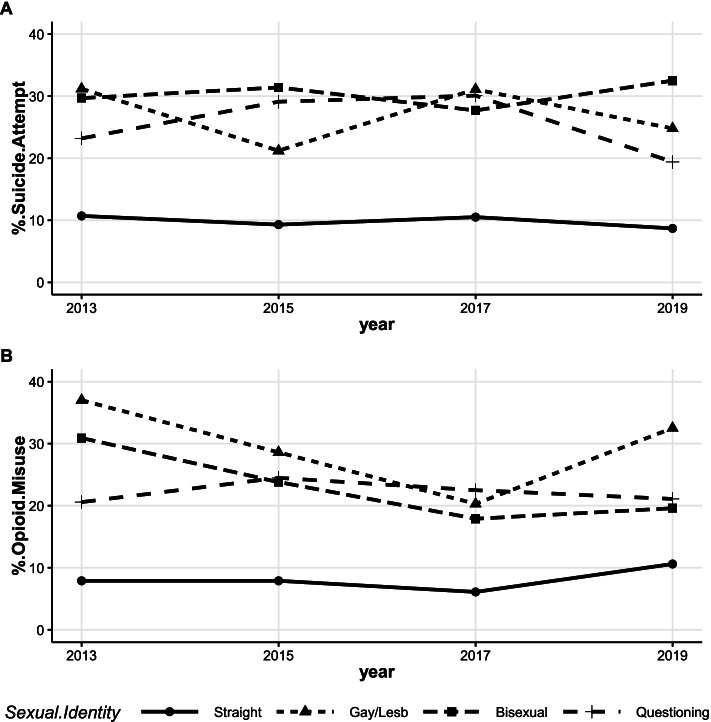
Prevalence of suicide attempt (**A**), and opioid misuse (**B**) among AI/AN youth by sexual orientation. Trend (**A**): straight (t = -1.20, *p*-value = 0.35), gay/lesbian (t = -0.36, *p*-value = 0.76), bisexual (t = -2.17, *p*-value = 0.16), questioning (t = -0.39, *p*-value = 0.73). Trend (**B**): straight (t = 0.69, *p*-value = 0.56), gay/lesbian (t = -0.69, *p*-value = 0.60), bisexual (t = -2.79, *p*-value = 0.11), questioning (t = -0.05, *p*-value = 0.96)

AI/AN high school students who identified as lesbian, gay, or bisexual had a higher prevalence of suicide attempt compared to heterosexual students during the period of the study (see Fig. 6A). No significant changes were observed in the suicide attempt prevalence trend over time among AI/AN students of any sexual orientation (straight: t = -1.20, *p*-value = 0.35; gay/lesbian: t = -0.36, *p*-value = 0.76; bisexual: t = -2.17, *p*-value = 0.16; questioning: t = -0.39, *p*-value = 0.73). The data showed a decline, however, in opioid misuse among youth who identified as gay/lesbian (37.0% in 2013 to 20.3% in 2017) and bisexual (30.9 in 2013 to 17.9 in 2017; Fig. 6B). There was a statistically significant decreasing trend among gay/lesbian AI/AN youth (t = -289.3, *p*-value = 0.002) from 2013 to 2017. Nevertheless, a sharp increase in the prevalence of opioid misuse occurred from 2017 to 2019 among gay/lesbian (20.3% ↑ 32.5%) and bisexual (17.9% ↑19.6%; Fig. 6B) AI/AN students.

**Fig. 7:**
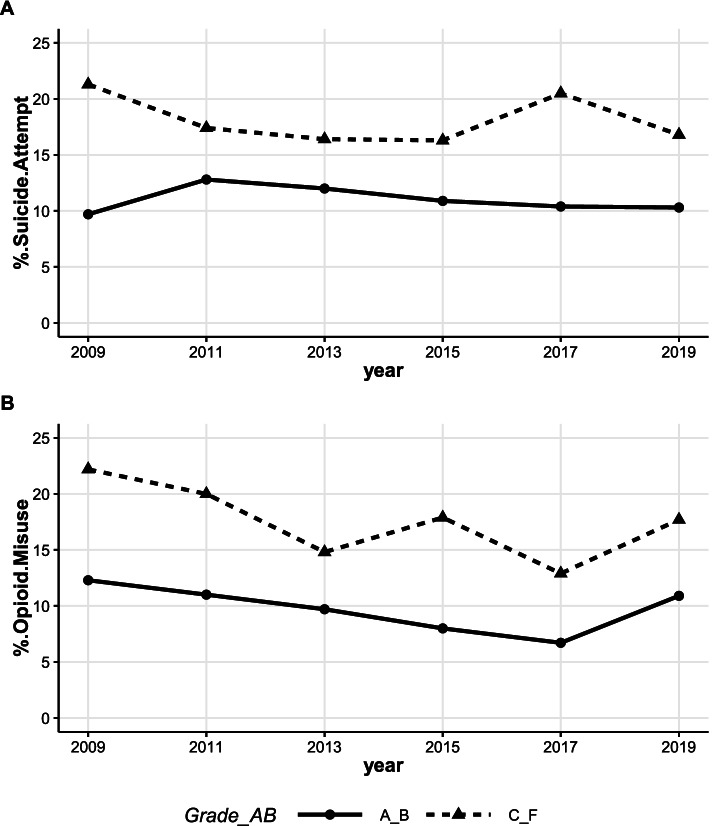
Prevalence of suicide attempt (**A**), and opioid misuse (**B**) among AI/AN stratified by academic performance. Trend (**A**): A_B (t = -0.50, *p*-value = 0.64), C_F (t = -0.68, *p*-value = 0.53). Trend (**B**): A_B (t = -1.33, *p*-value = 0.25 for all years, t = -35.5, *p*-value =  < .0001 for 2009 to 2017), C_F (t = -1.69, *p*-value = 0.17)

AI/AN high school students who had poor academic performance were also more likely to attempt suicide (21.3% in 2009 to 16.8% in 2019) than their counterparts who had excellent academic performance (9.7% in 2009 to 10.3% in 2019, Fig. 7A). Trend analysis showed no statistically significant changes in suicide attempt prevalence within these academic performance groups over time, high grades (A_B, t = -0.50, *p*-value = 0.64), poor grades (C_F, t = -0.68, *p*-value = 0.53). Furthermore, AI/AN students with poor academic performance were also more likely to misuse opioids than their counterparts with higher grades in school (22.2% in 2009 to 17.7% in 2019 for poor grades vs. 12.3% in 2009 to 10.9% in 2019 for high grades, Fig. 7B). Though AI/AN students with poor grades consistently had a higher prevalence of opioid misuse than those with higher grades (from 22.2% vs. 12.3% in 2009 to 12.9% vs. 6.7% in 2017), AI/AN students with both excellent and poor academic performance exhibited an increase in opioid misuse from 2017 to 2019 (6.7% ↑ 10.9% for high grades vs. 12.9%↑17.7% for poor grades). A significant decline in opioid misuse by students who had high grades was found (t = -35.5, *p*-value < 0.0001 for 2009 to 2017).

## Discussion

The objective of the current study was to determine and examine the prevalence of suicide attempt, opioid misuse, and social support among AI/AN high school students in New Mexico over time. We found that the prevalence of past-year suicide attempt over time varied by status of opioid misuse among AI/AN youth in New Mexico. These findings suggest opioid misuse has a significant association with suicide attempts for AI/AN high school students New Mexico. From 2009 to 2019, the prevalence of suicide attempt among New Mexico AI/AN high school students was consistently higher among those who misused opioids compared to those who did not misuse opioids. This finding is consistent with other studies [17, 31–33], which suggest that opioid misuse may be associated with increased risk of suicide in other populations.

Additionally, we found that AI/AN youth with high social support had the lowest prevalence of suicide attempt and opioid misuse. Conversely, youth with low social support had the highest prevalence of suicide attempt and opioid misuse. This finding corroborates recent research identifying social support as a protective factor for the risk of suicide attempt and opioid misuse [6, 24, 34–36], and highlights key factors communities can mobilize to decrease behavioral health risks among AI/AN youth.

Female AI/AN students were more likely to report attempted suicide than their male counterparts; however, male students were more likely to report opioid misuse than female students. This trend is consistent with other estimates of opioid misuse and suicide attempt in the extant literature [3]. Research has shown that women are more likely to attempt suicide; however, men are more likely to die by suicide [37–39] In addition, our study revealed a significant decreasing trend in opioid misuse (2009–2017) when stratified by gender. This finding mirors that of the state and could be linked to the long term impact of the screening, brief intervention, and referral to treatment (SBIRT) policy implemented by New Mexico in 2004 [40] and further, to making it mandatory in 2017 for healthcare providers to check a patient’s prescription history when prescribing opioids through the state’s Prescription Monitoring Program (PMP) database [41]. However, the sharp increase in 2019 is more likely due to the national increae in the use of synthetic opioid in recent years. The CDC reports that synthetic opioid-involved death rates increased by over 15% from 2018 to 2019, which accounted for almost 73% of all opioid-involved deaths in 2019 [42].

This study indicated that maternal education also might play a critical role in opioid misuse and suicide attempt. Recent studies conducted among AI/AN youth showed maternal education is a protective factor against suicide attempts [24] and opioid misuse [36]. Our analysis, along with previous studies, suggests that initiatives promoting women’s higher education in tribal communities and those targeting women of color may indirectly improve behavioral health outcomes among AI/AN youth.

AI/AN youth attending schools on non-tribal lands had a higher past-year suicide attempt and opioid misuse between 2011 through 2017 than their counterparts attending schools on tribal lands. Tribal communities may offer key protective factors and resources for resiliency relevant to AI/AN youth mental health outcomes. Some studies suggest the majority of AI/AN individuals who live on tribal communities have a lower probability of experiencing mental health distress, even with lower reported social support, than AI/AN individuals with low support who live on non-tribal [43, 44]. Similar results were seen in our study, as AI/AN youth attending schools in non-tribal lands had a higher prevalence of suicide attempt and opioid misuse. Strong cultural identity and familial ties among AI/AN youth in tribal communities may mediate opioid misuse and suicide attempt more than AI/AN youth in non-tribal lands.

Sexual minority AI/AN students exhibited disproportionately high prevalence of opioid misuse and suicide attempt than their heterosexual counterparts. A recent study examining the association between opioid misuse and suicidal behaviors among adolescents found that those who identified as lesbian, gay, or bisexual had a greater odds of suicide attempt when they misused opioids [45].

This study identified differences in suicide attempt and opioid misuse by AI/AN students with varied levels of academic performance. A recent study among AI adolescents on risk and protective factors for opioid misuse also demonstrated that poor school performance was associated with a greater likelihood of opioid misuse [46], suggesting that the association between behavioral health factors and academic outcomes is shared by additional youth populations beyond AI/AN populations in New Mexico.

This study provides a preliminary evaluation of trends in opioid misuse, social support, and suicide attempt among AI/AN youth populations. The analyses consider geographic, demographic, and socioeconomic variation in these trends, and examine data over 6-year period. The study’s limitations are the use of self-reported cross-sectional data, which makes it impossible to determine causation. The surveys also did not ask questions that may capture forms of cultural support relevant to AI/AN youth behavioral health outcomes, such as access to elders who speak their indigenous language or engagement in cultural practices like rites of passage. Also, the measurements of rurality and tribal land were specific to school’s location and not where the students reside allowing some contamination from those who commute from a rural area into an urban school setting, as an example. Despite these limitations, the survey data used in these analyses offer a larger AI/AN sample size than many youth surveys and state representative data. Thus, these results have the benefit of being generalizable to all AI/AN high school students in New Mexico.

## Conclusion

No significant trend was observed for suicide attempt. However, we observed a significant decreasing trend in opioid misuse between 2009 through 2017. We observed that high school students with high social support had lower prevalence of opioid misuse, and suicide attempt. This study provides tribes and the state of New Mexico with state-level evidence of the trends in opioid misuse, social support, and suicide attempt among AI/AN high school students, and offers essential background information for community health efforts to reduce these public health problems. We recommend that more resources would be channeled into behavioral health care services for AI/AN youth, and provide funding and other relevant resources to support teen centers and school-based health centers in New Mexico.

## Data Availability

The datasets generated during and/or analyzed during the current study are not publicly available due to restrictions by the New Mexico Department of Health (NMDOH), and the Albuquerque Area Southwest Tribal Epidemiology Center (AASTEC); the providers of the AI/AN oversampled NMYRRS data. Data could be accessed by signing a data sharing agreement with NMDOH and AASTEC (Dr. Kevin English: https://www.aastec.net/).
